# Tubal Patency Decoction and Its Ingredient β‐Sitosterol Alleviate Tubal Infertility by Inhibiting Inflammation and Promoting Cilia Formation Through PGR

**DOI:** 10.1155/mi/8896503

**Published:** 2026-07-27

**Authors:** Liang Shao, Nansu Wang, Yan Yan, Qiongfang Tan, Yuying Huang, Qin Wu, Yali Tan, Yue Zhu, Yaqiong Xia, Ling Liu

**Affiliations:** ^1^ Department of Emergency, The First Affiliated Hospital of Hunan Traditional Chinese Medicine College, Zhuzhou, Hunan, China; ^2^ Department of TCM Gynaecology, The First Affiliated Hospital of Hunan Traditional Chinese Medicine College, Zhuzhou, Hunan, China; ^3^ Graduate School, Hunan University of Chinese Medicine, Changsha, Hunan, China, hnctcm.edu.cn

**Keywords:** β-sitosterol, ciliogenesis, inflammation, tubal infertility, tubal patency decoction (TPD)

## Abstract

**Background:**

Tubal infertility is one of the major causes of female infertility. This study aimed to investigate the mechanism of action of tubal patency decoction (TPD) and its active ingredient β‐sitosterol on tubal infertility.

**Methods:**

The active ingredients of TPD and their potential targets were obtained from the TCMSP and Batman‐TCM databases. The GeneCards, NCBI, and OMIM databases provided the targets associated with tubal infertility. The relationship between the components of TPD and the potential targets of tubal infertility was analyzed by String database and Cytoscape 3.8.0 software. The composition of the compounds in TPD was examined by liquid chromatography‐mass spectrometry (LC‐MS) assay. The effects of TPD and β‐sitosterol on tubal infertility were verified by establishing a rat model of tubal infertility and a model of tubal epithelial cell inflammation.

**Results:**

By network pharmacological analysis, we screened 139 active ingredients of TPD, covering 523 targets, of which 34 were related to tubal infertility. β‐sitosterol was identified as an important active compound. Progesterone receptor (PGR) was considered the main target of TPD and β‐sitosterol in tubal infertility. Molecular docking and drug affinity responsive target stability (DARTS) assays showed that PGR binds to β‐sitosterol. The results of cellular experiments confirmed that β‐sitosterol and TPD‐containing serum increased the cell viability of LPS‐induced tubal epithelial cells, decreased the levels of inflammatory factors (IL‐1β, IL‐6, IL‐8, and TNF‐α) and myeloperoxidase (MPO), and increased the frequency of ciliary beating. The results of in vivo experiments showed that both TPD and β‐sitosterol improved the conception rate and live births in tubal infertile rats and attenuated tubal inflammation and epithelial cell damage. They increased the expression of RFX2, RFX3, and FOXJ1. Silencing PGR reversed the effect of β‐sitosterol on tubal epithelial cells and tubal infertile rats.

**Conclusion:**

TPD and its active ingredient β‐sitosterol may improve tubal infertility through PGR. These results provide a new basis for the development and application of TPD and its active ingredient β‐sitosterol.

## 1. Introduction

The failure of a couple to conceive after 12 months of regular, unprotected sex in the reproductive age group is defined as infertility [1]. Ovulatory dysfunction, male factor infertility, and tubal disease are the main causes of infertility, with tubal factors accounting for approximately 25% of infertility cases [2, 3]. The fallopian tubes help transport sperm from the isthmus to the ampulla for fertilization. They also provide a microenvironment through secretions and signaling that supports not only the survival and development of ovulated/fertilized eggs and pre‐implantation embryos but also the survival and development of sperm, among other things [4]. Tubal infertility is defined as obstruction of the fallopian tubes or the inability to take oocytes from the ovaries due to pelvic adhesions [2, 5]. Conventional treatment for tubal infertility includes gynecological endoscopic surgery and interventional recanalization [6, 7]. Selective salpingography is performed using a coaxial catheter on a guide wire, followed by interventional recanalization of the tubal obstruction. However, due to its complex pathology, occlusion of the tubal lumen after the procedure is likely to occur, resulting in a low rate of post‐treatment intrauterine pregnancy [8, 9]. Therefore, the development of new therapeutic tools is necessary.

Transport in the fallopian tube is driven by two main mechanisms, smooth muscle contraction and ciliary movement, which play a dominant role in the transport of fertilized eggs [10]. Synchronized beating of the cilia generates the driving force to move fluid and oocytes through the fallopian tube [11]. Lack of cilia and ciliary dysfunction in the tubal epithelium may lead to female infertility [12, 13]. Inflammation of the fallopian tube has also been shown to be an important cause of infertility in humans and animals [14]. It is important to study tubal inflammation and cilia formation to better understand and improve tubal infertility.

Traditional Chinese medicine (TCM) classifies tubal infertility into Qi stagnation and blood stasis syndrome, stasis, and water stagnation syndrome, cold and damp stasis syndrome, Qi deficiency and blood stasis syndrome, and damp‐heat stasis syndrome [15, 16]. Compared with western fertility medication, TCM treatment of female infertility can increase the pregnancy rate by two times within 3–6 months [17]. In addition, TCM has been used as an adjunctive therapy for tubal recanalization, which not only shortens the treatment time but also prevents re‐obstruction and improves pregnancy rates [15, 18, 19]. Tubal patency decoction (TPD) is designed based on the TCM principle of “activating blood circulation, resolving stasis, dredging collaterals, and dispersing stagnation,” integrated with modern medical understanding of tubal obstruction mechanisms (such as inflammatory responses) [20]. It is composed of Danggui (*Angelicae Sinensis Radix*), Shengdi (*Rehmannia glutinosa*), Taoren (*Persicae Semen*), Honghua (*Carthami Flos*), Chishao (*Radix Paeoniae Rubra*), Chaihu (*Radix Bupleuri*), Chuanxiong (*Chuanxiong Rhizoma*), Gancao (*licorice*), Shuizhi (*Hirudo*), Lulutong (*Fructus Liquidambaris*), Sigualuo (*Luffae Fructus Retinervus*), Zaojiaoci (*Gleditsiae Spina*), Chuanlianzi (*Toosendan Fructus*), and Jixueteng (*Spatholobus Suberectus Dunn*). In the TPD formula, components—such as Taoren, Honghua, and Chuanxiong—possess the effects of promoting blood circulation and removing stasis, while Lulutong, Sigualuo, and Zaojiaoci exhibit the actions of activating blood circulation, dredging collaterals, resolving water retention, and dispersing stagnation. Our previous animal experiments confirmed that TPD regulates the JAK2/STAT3 inflammatory signaling pathway in the rat fallopian tube tissue, significantly reduces IL‐6 and TNF‐α levels in rat models of tubal infertility, and ameliorates inflammatory conditions in the fallopian tubes [21]. However, the precise mechanism of TPD in treating tubal infertility remains unclear.

Due to the characteristics of Chinese medicine compounds involving multiple components and multiple targets, it is difficult to systematically elucidate their overall action characteristics by traditional research methods [22]. With the development of bioinformatics, the emergence of network pharmacology provides a new direction for the study of Chinese medicine compounding [23]. Network pharmacology is an effective method to systematically and comprehensively reveal the bioactive components of TCM compounding and their relationship with potential targets [24]. We used network pharmacology to screen the key components of TPD and their targets of action on tubal infertility. By constructing a rat model of tubal infertility and a tubal epithelial cell inflammation model, we explored the effects and mechanisms of action of TPD and its key components on tubal infertility, intending to provide new clues and research bases for the treatment of this disease.

## 2. Materials and Methods

### 2.1. Preparation of TPD

The Pharmacy Department of the First Affiliated Hospital of Hunan TCM College identified the varieties and sources of the same batch of Chinese herbs. Danggui (*Angelicae Sinensis Radix*) 15 g, Shengdi (*Rehmannia glutinosa*) 15 g, Taoren (*Persicae Semen*) 15 g, Honghua (*Carthami Flos*) 10 g, Chishao (*Radix Paeoniae Rubra*) 10 g, Chaihu (*Radix Bupleuri*) 10 g, Chuanxiong (*Chuanxiong Rhizoma*) 10 g, Gancao (*licorice*) 6 g, Shuizhi (*Hirudo*) 3 g, Lulutong (*Fructus Liquidambaris*) 15 g, Sigualuo (*Luffae Fructus Retinervus*) 12 g, Zaojiaoci (*Gleditsiae Spina*) 12 g, Chuanlianzi (*Toosendan Fructus*) 10 g, and Jixueteng (*Spatholobus Suberectus Dunn*) 15 g. The daily dose for humans was 160 g, and the equivalent dose for rats was 16.7 g/kg (the middle dose group), with the high dose group at 33.4 g/kg and the low dose group at 8.4 g/kg.

### 2.2. Tubal Infertility Model

A model of tubal infertility was established following previous studies [25]. The modeling method was based on Professor Ma Baozhang’s “Mixed Bacteria Inoculation Method,” which has been replicated in several papers and has been proven to cause typical tubal inflammatory hydrops and obstruction. SPF‐grade SD rats (250–300 g, 8–10 weeks old) were routinely observed for 1 week. Modeling was carried out following the mixed bacterial method. Sodium pentobarbital (50 mg/kg) was administered intraperitoneally to rats to induce anesthesia. After the anesthesia took effect, the rats were secured on the operating table with fine cotton thread. The abdomen was depilated, the skin was prepared for surgery, and then disinfected with complex iodine. A median incision of about 0.8–1 cm was made in the lower abdomen. After entering the abdominal cavity, the bicornuate uterus was identified, and the bilateral fallopian tubes were exposed. *Escherichia coli*, *Staphylococcus aureus*, and *Streptococcus* were mixed in a ratio of 2 : 1:1 in sterile saline to form a mixed bacterial suspension at a concentration of 3 × 10^9^ CFU/mL. Using a 1 mL syringe, 0.1 mL of the suspension was injected into each oviduct by inserting the needle into the uterine horn near the oviduct and directing it toward the ovary. In the sham group, after the same incision was made, the abdominal wall was sutured, and an appropriate bandage was applied (without bacterial injection).

### 2.3. Animal Grouping and Treatment

To investigate the effect of TPD on tubal infertility, SD rats were randomly divided into Sham, Model, LTPD, MTPD, and HTPD groups. Rats in the Model, LTPD, MTPD, and HTPD groups were used to establish the tubal infertility model. The Sham and Model groups were treated with a gavage of distilled water. The LTPD group, MTPD group, and HTPD group were gavaged with TPD at doses of 8.4, 16.7, and 33.4 g/kg, respectively, once daily in the morning for 28 consecutive days [25].

To investigate the effects of β‐sitosterol and progesterone receptor (PGR) on tubal infertility, SD rats were randomly divided into Sham, Model, β‐sitosterol, β‐sitosterol + sh‐NC, and β‐sitosterol + sh‐PGR groups. The Model, β‐sitosterol, β‐sitosterol + sh‐NC, and β‐sitosterol + sh‐PGR groups were established as tubal infertility models. The Sham and Model groups were gavaged with distilled water. β‐sitosterol group, β‐sitosterol + sh‐NC group, and β‐sitosterol + sh‐PGR group rats were administered β‐sitosterol (S24012, Yuanye Biotechnology Co., Ltd., China) 200 mg/kg by gavage once a day for 3 weeks [26]. Rats in the β‐sitosterol + sh‐NC group and rats in the β‐sitosterol + sh‐PGR group were simultaneously given approximately 10 μL of lentiviral vector solution (sh‐PGR and its sh‐NC, at a titer of 2 × 10^8^ TU/mL) by slow microinjection into the subperitoneal space of the ovary [27].

Mature male rats were randomly assigned to groups of two rats each, and each group was housed with 5–7 female rats from the corresponding group in the same cage. The conception rate was calculated after 10 days.

### 2.4. Hematoxylin and Eosin Staining (HE Staining)

After fixation in paraformaldehyde and embedding in paraffin, the tubal tissues were sliced into 4 μm sections. Following 12 h of baking at 60°C, the slices were dewaxed and rehydrated and then stained with HE (G1125, Beijing Solarbio Science and Technology Co., Ltd., China) for 5 min. The slices were dehydrated with a gradient of alcohol (95%–100%) and then treated with xylene for 10 min. Sections were sealed with neutral gum (AWI0238a, Abiowell), and the results were observed under a light microscope (BA210T, Motic, China).

### 2.5. Electron Microscopic Observation of Oviduct Ultrastructure

Tubal tissues were prefixed with 2.5% glutaraldehyde (G5882, Sigma–Aldrich, USA). After rinsing three times with 0.1 M phosphate buffer, the tubal tissues were fixed again with 1% osmium acid (AWI0136, Abiowell). The fixed tubal tissues were dehydrated stepwise in a graded series of acetone (50%, 70%, 80%, 90%, and 100%). The tissues were infiltrated with a 1:1 mixture of Epon812 embedding solution (GS02659, Beijing Zhongjing Science and Technology Co., Ltd., China) and acetone for 30 min. Then, the tissues were immersed in pure Epon812 for 1 h, followed by polymerization at 37°C for 24 h and then at 60°C for 48 h. Ultrathin sections of 60–90 nm were made with an ultrathin sectioning machine and floated onto a copper mesh. The sections were stained with uranyl acetate (GZ02625, Beijing Zhongjing Science and Technology Co., Ltd.) for 10–15 min and then with lead citrate (GA10701‐1, Beijing Zhongjing Science and Technology Co., Ltd.) for 1–2 min, and finally, the ultrastructure of the tubal tissues was observed under an electron microscope.

### 2.6. Detection of IL‐1β, TNF‐α, IL‐6, IL‐8, and Myeloperoxidase (MPO)

The levels of IL‐1β, TNF‐α, IL‐6, and IL‐8 in serum and cell supernatants were determined following the guidelines provided by the respective enzyme‐linked immunosorbent assay (ELISA) kits: IL‐1β (JL20884, Jonlogy Co., Ltd., China), TNF‐α (CSB‐E11987r, CUSABIO, China), IL‐6 (CSB‐E04640r, CUSABIO), and IL‐8 (ml002885, Mlbio, China). MPO levels in tissues and cells were detected according to the procedure of the biochemical kit (A044‐1‐1, Nanjing Jiancheng Bioengineering Institute, China).

### 2.7. Reverse Transcription‐Quantitative Polymerase Chain Reaction (RT‐qPCR)

Total RNA was extracted from tissues and cells using the Trizol reagent (15596026, Thermo, USA). cDNAs were synthesized by applying an mRNA Reverse Transcription Kit (CW2569, CWBIO, China). cDNAs were prepared by qPCR reaction in a fluorescent quantitative PCR system (PIKOREAL96, Thermo) according to the UltraSYBR Mixture (CW2601, CWBIO). The 2^−ΔΔCt^ method was used to calculate the relative expression levels with GAPDH as an internal reference. Primer sequences are shown in Table 1.

**Table 1:** Primer sequences.

Gene	Sequences (5′–3′)
GAPDH	F: ACAGCAACAGGGTGGTGGAC
	R: TTTGAGGGTGCAGCGAACTT
RFX2	F: AGACTGTAGCCGTGGAGACT
	R: GCATGCTGGGATCTGGAGTT
RFX3	F: CACCATCTCCATCACCCACC
	R: CCTGTGTCTGAACCCGTCTC
FOXJ1	F: AGCGTCTACTTAATGCCGGTC
	R: GAAGAAGTTCCTCCCACCTC
PGR	F: CTTCCCAGACTGCACCTACC
	R: AGGCTGGAATTCGCCGTAAA

### 2.8. The Target Information of the Ingredients of TPD

The compound components and targets of Danggui, Shengdi, Taoren, Honghua, Chishao, Chaihu, Chuanxiong, Gancao, Shuizhi, Lulutong, Sigualuo, Zaojiaoci, Chuanlianzi, and Jixueteng were searched through the TCMSP database [28] (https://tcmspw.com/tcmsp.php), with the screening criteria of OB ≥ 30% and DL ≥ 0.18. Since the TCMSP database does not include Shengdi, Shuizhi, and Sigualuo, compound components of these three were supplemented using the Batman‐TCM database [29] (http://bionet.ncpsb.org.cn/batman-tcm/), with a selection of components and targets with a score cutoff ≥ 0.89. All targets were standardized using the UniProt database [30] (https://www.uniprot.org/) to exclude non‐human targets. After summarizing and removing duplicates, the numbers of compounds and targets for each herb were as follows: Gancao had 93 compounds and 216 targets; Chaihu had 17 compounds and 179 targets; Chishao had 29 compounds and 94 targets; Chuanlianzi had nine compounds and 144 targets; Chuanxiong had seven compounds and 30 targets; Danggui had two compounds and 51 targets; Honghua had 22 compounds and 203 targets; Jixueteng had 24 compounds and 127 targets; Lulutong had four compounds and 55 targets; Taoren had 23 compounds and 50 targets; Zaojiaoci had 11 compounds and 200 targets; Shengdi had 21 compounds and 195 targets; Shuizhi had 16 compounds and 228 targets; and Sigualuo had four compounds and 48 targets.

### 2.9. Disease Targets

Using “fallopian tube inflammatory infertility,” “tubal factor infertility,” and “tubal infertility” as keywords, human gene searches were performed in the GeneCards database [31] (https://www.genecards.org/), NCBI gene database [32] (https://www.ncbi.nlm.nih.gov/), and OMIM database [33] (https://www.omim.org/). After combining and deleting genes from these three databases, 710 disease‐related genes were obtained. The screened drug targets and disease‐related genes were entered into Venn diagram creation software Venny 2.1, and 34 common targets were obtained.

### 2.10. PPI Network Construction

“*Homo sapiens”* was selected as the biological species, and the drug‐disease common targets were entered into the String database [34] (https://string-db.org/cgi/input.pl) to create the PPI network.

### 2.11. Topology Analysis

After importing the PPI network into Cystoscape 3.8.0 [35], the NetworkAnalyzer tool conducted a topology analysis, and genes with scores higher than the average score were chosen as key targets using degree sorting.

### 2.12. Liquid Chromatography‐Mass Spectrometry (LC‐MS)

In accordance with earlier research, we employed LC‐MS to identify the chemical makeup of TPD [36]. Briefly, 1 mL of methanol:ethyl ether:water (2:2:1) was mixed with 100 mg of TPD. After 10 min of precipitation, the mixture was sonicated at 4°C. To extract the supernatant, the mixture was centrifuged after being kept at −20°C for 1 h. A cryo‐concentrator was then used to remove the supernatant, and 200 μL of acetonitrile:water (1:1) was added to re‐dissolve it. The LC‐MS apparatus (LC‐MS–MS‐8050, Shimadzu, Japan) was used to examine the reconstituted samples. A Waters HSS T3 column (100 × 2.1 mm, 1.7 µm) was used as the chromatographic column. The injection volume was 3 μL, and the mobile phases A and B were acetonitrile and 0.1% HCOOH–H_2_O, respectively. The data were analyzed using MS‐DIAL 4.10 software [37].

### 2.13. Isolation, Culture, and Identification of Rat Oviduct Epithelial Cells

Rats were sacrificed via neck‐breaking. The oviducts were removed and placed in 5 mL of HBSS, and the fat and ovaries were removed from the tissue under a dissecting microscope. The oviducts were washed with HBSS on an ultraclean table, and the tissues were cut into mince with ophthalmic scissors. The cut tissues were transferred to culture flasks containing 0.25% trypsin and enzymatically digested for 30 min in a water shaker at 37°C. The mixture was transferred to a centrifuge tube and centrifuged at 1000 r/min for 5 min. The supernatant was discarded, and the tissues were transferred to the culture flasks containing 1 mg/mL collagenase (HBSS) and enzymatically digested for 30 min in a water shaker at 37°C. The mixture was transferred to the centrifuge tube and centrifuged at 1000 r/min for 5 min. The supernatant was discarded, and 1 mL of DMEM/F‐12 medium was added to create a cell suspension by blowing gently. The cell suspension was transferred to disposable flasks and cultured at 37°C and 5% CO_2_ for about 4 h. Then, the upper layer of cell suspension was sucked out and transferred into a centrifuge tube and centrifuged for 5 min at 1000 *r*/min. The supernatant was discarded, and then, 1 mL of DMEM/F‐12 medium was added to the cell suspension. The suspension was then inoculated into a disposable petri dish and cultivated in a 37°C, 5% CO_2_ incubator. The medium was changed every 48 h. When the rat oviduct epithelial cells grew to 80% confluence or spread all over the bottom of the dish, they could be passaged.

Immunofluorescence staining was used to identify the epithelial cells [38]. Immunofluorescence staining showed that the cells expressed cytokeratin 18 (10830‐1‐AP, Proteintech, USA) and did not express vimentin (AWA10146, Abiowell), confirming that the isolated cells were tubal epithelial cells.

### 2.14. Preparation of Medicated Serum [39]

SD rats received the following treatments to collect TPD‐containing serum: intragastric administration of normal saline (blank serum group), intragastric administration of 8.4 g/kg TPD (LTPD group), 16.7 g/kg TPD (MTPD group), and 33.4 g/kg TPD (HTPD group). All administrations were performed at the same time each morning, once daily for 28 consecutive days. One hour after the final administration, rats were anesthetized by intraperitoneal injection of 2% sodium pentobarbital solution (40 mg/kg). Blood samples were collected from the abdominal aorta under aseptic conditions. The collected blood was centrifuged at 3000 rpm for 15 min to separate the serum. The supernatant was heat‐inactivated (56°C, 30 min) and finally filtered through a 0.22 μm membrane. The filtered serum was aliquoted and stored at −80°C for subsequent experiments.

### 2.15. Treatment of Rat Oviduct Epithelial Cells

The above‐extracted rat oviduct epithelial cells were selected and stimulated for 24 h using 200 ng/mL LPS to induce a cellular inflammation model.

To verify the effect of TPD on oviduct epithelial cells, the cells were treated with the corresponding TPD‐containing serum for 24 h. The six experimental groups included the control group, LPS group, blank serum group, LTPD group, MTPD group, and HTPD group. Cells in the control group were cultured under normal conditions. Cells in the LPS group were stimulated with 200 ng/mL LPS for 24 h. Cells in the blank serum group, LTPD group, MTPD group, and HTPD group were simultaneously stimulated with 200 ng/mL LPS and treated with the corresponding drug‐containing serum for 24 h.

To examine the impact of PGR on oviductal epithelial cells, the cells were divided into control, LPS, si‐NC, si‐PGR, oe‐NC, and oe‐PGR groups. Cells in the control group were cultured normally. Cells in the LPS group were stimulated with 200 ng/mL LPS for 24 h. Cells in the si‐NC, si‐PGR, oe‐NC, and oe‐PGR groups were transfected with Lipofectamine 2000 reagent (11668‐019, Invitrogen) for 48 h with si‐PGR, oe‐PGR, and their corresponding blank plasmids, respectively, and then stimulated for 24 h with 200 ng/mL LPS.

To investigate the effect of β‐sitosterol on rat oviduct epithelial cells, they were treated with 0, 1, 2, and 5 μM β‐sitosterol for 24 h, respectively.

To investigate whether β‐sitosterol and PGR affected LPS‐induced tubal epithelial cell injury, the cells were divided into control, LPS, β‐sitosterol, β‐sitosterol + si‐NC, and β‐sitosterol + si‐PGR groups. The control group’s cells were cultivated in a standard manner. The LPS group’s cells were cultivated with 200 ng/mL LPS for 24 h. The cells in the β‐sitosterol group were treated with 2 μM β‐sitosterol for 24 h after being stimulated with 200 ng/mL LPS for 24 h. The β‐sitosterol + si‐NC group’s cells were transfected with si‐NC, stimulated for 24 h with 200 ng/mL LPS, and then treated for 24 h with 2 μM β‐sitosterol. The β‐sitosterol + si‐PGR group’s cells were transfected with si‐PGR, stimulated with 200 ng/mL LPS for 24 h, and then treated with 2 μM β‐sitosterol for 24 h.

### 2.16. Molecular Docking and Drug Affinity Responsive Target Stability (DARTS) Assay

Molecular docking was utilized to investigate the binding of β‐sitosterol to PGR. The compounds and proteins were analyzed through a docking study using VINA 1.1.2 software, which employs a semi‐empirical free energy field to estimate the binding energies of ligands and receptors. Following this, PYMOL was employed to assess the stable binding of the compounds within the protein’s cavity and their interactions with the surrounding amino acids.

The DARTS assay was performed according to the procedure described previously [40, 41]. Briefly, rat oviduct epithelial cells were collected and lysed. Cell lysates were treated or not with β‐sitosterol (1, 2, and 5 μM). After incubation, each 300 μg of lysate was lysed with 1 μg of Pronase for 30 min. Protein loading buffer was added immediately and boiled for 5 min for western blot analysis.

### 2.17. Western Blot Analysis

Total cellular proteins were extracted with the RIPA lysate (P0013B, Beyotime Biotechnology). The protein concentration was determined according to the BCA protein quantification kit instructions. The same mass of protein was taken from each group and loaded onto a bis–tris gel. After electrophoresis, the proteins were transferred to the membrane. The membranes were immersed in a 5% blocking solution and blocked at room temperature for 1.5 h. An appropriate amount of primary antibody PGR (ab32085, Abcam, UK) and GAPDH (10494‐1‐AP, Proteintech) was added to each group and was incubated at 4°C overnight. A secondary antibody HRP goat anti‐rabbit IgG (SA00001‐2, Proteintech) was added and reacted at room temperature for 1 h. The target bands were visualized by SuperECL Plus ultrasensitive luminescent solution (K‐12045‐D50, Advansta, USA) and imaged with a gel imaging system (ChemiScope6100, Clinx, China). GAPDH was used as an internal reference.

### 2.18. Cell Counting Kit‐8 (CCK8) Assay for Cell Viability

Cell viability was determined using CCK8, and cells were placed in 96‐well plates at a density of 8 × 10^3^ cells/well. After treating the cells in groups as described above, 10 μL of CCK‐8 solution was added to each well and then incubated at 37°C for 4 h. Cell viability was assessed by measuring the optical density (OD) at 450 nm using a microplate reader (MB580, HEALES, China).

### 2.19. Ciliary Beating Frequency Assay

To observe the ciliary beating frequency of cultured ciliated epithelial cells, a coverslip was removed from the culture plate and placed in a specific dosing tank on the microscope stage. The sHBSS was added, and the volume of the dosing tank was about 400 μL. The observation was carried out by applying an Olympus IX71 inverted phase contrast microscope under an oil microscope (Uplan Apo40XOI3Ph) at a magnification of 400 times. The phase contrast images were captured through a high‐speed digital camera acquisition system, that is, PULNiX high‐speed digital video camera, into a minicomputer workstation and through a collection procedure (Stream Pix 3.10.0), which could capture 240 frames/s of phase contrast images in sequence format, with a size of 648 pixels × 200 lines, which were then converted into avi format files. They were continuously captured for 3 s at a time. The images were analyzed by a specific software system, IPLab V3.65, on a 3 × 3‐pixel area in the mid‐section of the cilia to calculate the frequency of cilia oscillations by analyzing the changes in gray values in the selected area.

### 2.20. Data Analysis

Statistical analyses and graph preparation were performed using GraphPad Prism 9 software. Data were presented as means ± standard deviations. One‐way ANOVA followed by Tukey’s multiple comparison test was used to evaluate group differences. A *p*‐value <0.05 was considered statistically significant.

## 3. Results

### 3.1. TPD Can Alleviate the Symptoms of Tubal Infertility in Rats

**Figure 1:**
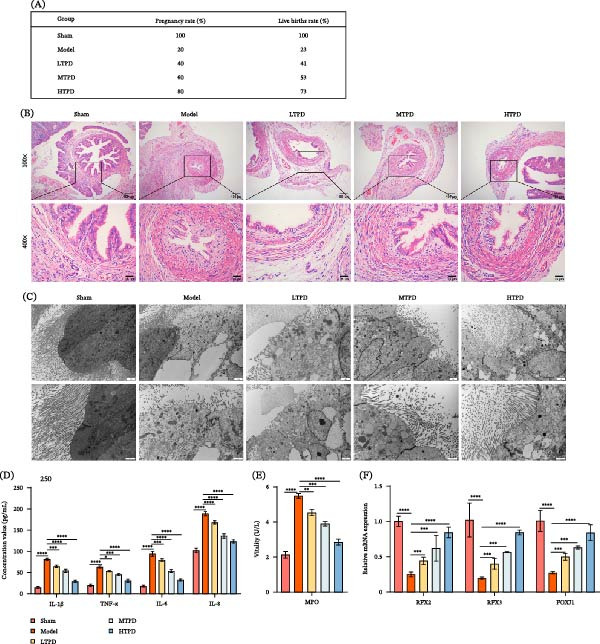
TPD can alleviate the symptoms of tubal infertility in rats. (A) Conception rate and live birth rate statistics. (B) HE staining. (C) Electron microscopy. (D) ELISA to detect serum IL‐1β, IL‐6, IL‐8, and TNF‐α. (E) MPO levels. (F) RT‐qPCR to detect the expression of cilia‐forming related factors, such as RFX2 and RFX3, and the main regulator of cilia movement, FOXJ1. Expression of FOXJ1. *n* = 5 rats per group.  ^∗^ *p*  < 0.05,  ^∗∗^ *p*  < 0.01,  ^∗∗∗^ *p*  < 0.001, and  ^∗∗∗∗^ *p*  < 0.0001.

To investigate the effect of TPD on tubal infertile rats, a model of tubal infertility was established. Then, different concentrations of TPD were used to intervene. Compared with the Sham group, the conception rate and live birth rate were significantly lower in the Model group (Figure 1A). Compared with the rats in the Model group, the conception rate and live birth rate of rats with low, medium, and high concentrations of TPD were significantly higher, especially in the high concentration group. The HE staining of the Sham group showed that the columnar epithelial cells were abundant and regular (Figure 1B). In contrast, that of the Model group showed that the epithelial cells were detached and there was severe epithelial damage. Electron microscopy showed that the epithelial cells in the Sham group had long cilia and dense microvilli (Figure 1C). In contrast, in the Model group, there were no obvious cilia and microvilli in the bare and necrotic epithelial area, and the stomata could be seen to ooze out globules. These symptoms were relieved after TPD treatment, and the effect was most pronounced in the high‐concentration group. In addition, the levels of IL‐1β, IL‐6, IL‐8, TNF‐α, and MPO were increased in the Model group relative to the Sham group and decreased after the treatment of TPD (Figure 1D,E). The expression of RFX2 and RFX3, which are related to the formation of cilia, as well as the expression of FOXJ1, which is the main regulator of cilia movement, was down‐regulated in the Model group and up‐regulated after the treatment of TPD (Figure 1F). These results show that TPD treatment alleviates the symptoms of tubal infertility in rats.

### 3.2. TPD Can Inhibit Tubal Epithelial Cell Inflammation and Increase Cell Viability and Ciliary Beating Frequency

**Figure 2:**
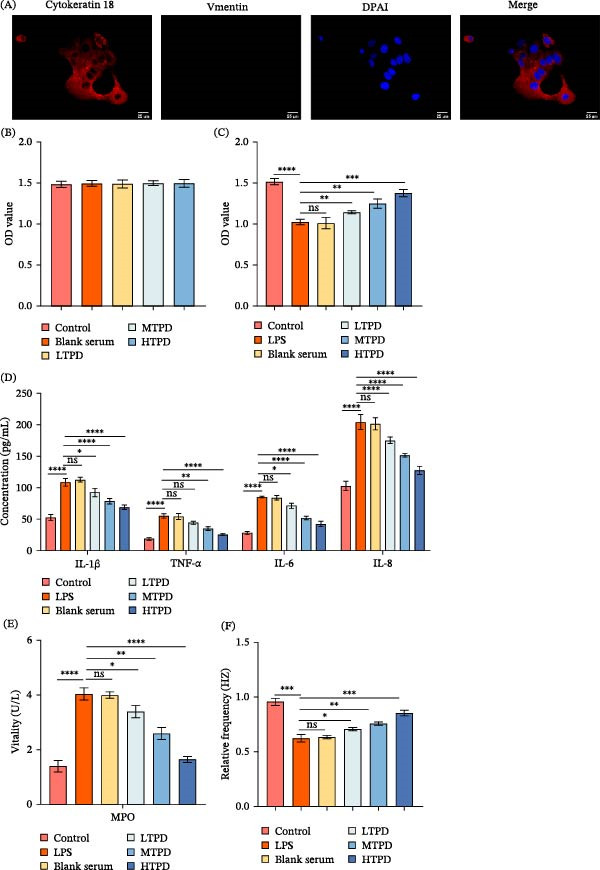
TPD can inhibit tubal epithelial cell inflammation and increase cell viability and ciliary beating frequency. (A) Immunofluorescence staining for cytokeratin 18 and vimentin to identify tubal epithelial cells. (B) Normal cells were treated with or without TPD‐containing serum, and cytotoxicity was detected by CCK8. (C) Cell viability was detected by CCK8. (D) ELISA was performed to detect IL‐1β, IL‐6, IL‐8, and TNF‐α in the cell supernatant. (E) Biochemical assay was performed to detect the level of MPO. (F) Detection of ciliary beating frequency. *n* = 3 biological replicates.  ^∗^ *p*  < 0.05,  ^∗∗^ *p*  < 0.01,  ^∗∗∗^ *p*  < 0.001, and  ^∗∗∗∗^ *p*  < 0.0001. ns, no significant difference.

To investigate the mechanism by which TPD affects tubal epithelial cell inflammation, we isolated tubal epithelial cells from rat fallopian tubes. Immunofluorescence staining showed that the cells expressed cytokeratin 18 and did not express vimentin, confirming that the isolated cells were tubal epithelial cells (Figure 2A). We examined the cytotoxicity of the TPD‐containing serum and found that it had no adverse effects on cell viability (Figure 2B). Further experiments demonstrated that treatment with the TPD‐containing serum significantly enhanced cell viability in LPS‐induced cells in a dose‐dependent manner (Figure 2C). Additionally, the TPD‐containing serum significantly suppressed the LPS‐induced elevation of inflammatory cytokine levels and MPO activity (Figure 2D,E). Moreover, it markedly increased the ciliary beating frequency of LPS‐treated cells (Figure 2F). Among all groups, the high‐dose group showed the most significant effects. The aforementioned data suggest that TPD could inhibit LPS‐induced damage and inflammation in the fallopian tube epithelial cells.

### 3.3. Network Pharmacology and LC‐MS Analyses of Key Components of TPD for the Treatment of Tubal Infertility

In order to investigate the key mechanism of TPD in alleviating tubal infertility, we analyzed the common targets of action of TPD and tubal infertility by network pharmacology. The Venn diagram showed a total of 34 potential targets of action for TPD and tubal infertility (Figure 3A). The drug‐disease common targets were subjected to the construction of a PPI network (Figure 3B). The network graph had 34 nodes, 292 edges, and an average degree value of 17.2. The PPI network was imported into Cystoscape 3.8.0 [35], topologically analyzed by the NetworkAnalyzer tool, and sorted by degree. All the targets were pictured using R 4.1.2, with the horizontal coordinates being the degree value of each target (Figure 3C). To better understand the intricate interactions between components, diseases, and their related targets, we constructed a component‐disease‐target network diagram using the included components, therapeutic diseases, and targets of action (Figure 3D). The ingredient‐disease‐target network diagram was topologically analyzed, and then, the 136 active compounds were ranked by degree. The higher the degree value, the more important the ingredient. The top five active ingredients in terms of TPD are listed in Table 2, including quercetin, 3beta‐hydroxyurs‐12‐en‐28‐syre, ursolic acid, β‐sitosterol, and luteolin. β‐sitosterol is a natural steroidal compound with a structure highly similar to cholesterol and exhibits estrogenic activity [42]. Studies have shown that β‐sitosterol suppresses endometrial cell proliferation by modulating the Smad7‐mediated TGF‐β/Smads signaling pathway [43]. Therefore, β‐sitosterol was selected as the candidate compound for further investigation in this study. We observed the presence of β‐sitosterol in TPD by LC‐MS (Figure 3E).

Figure 3Network pharmacology and LC‐MS analyses of key components of TPD for the treatment of tubal infertility. (A) Venn diagram showing the potential targets of action of TPD and tubal infertility. (B) The PPI network of key target genes of TPD and tubal infertility. (C) Topological analysis showing the key gene targets. (D) Active ingredient‐disease‐pathway‐target network diagram. (E) LC‐MS detection of the key ingredient β‐sitosterol in TPD.

**Figure:**
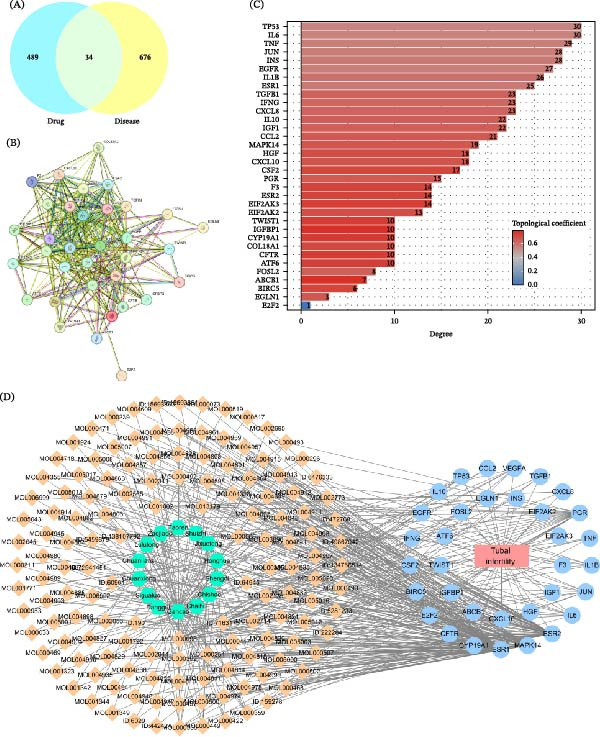

**Figure:**
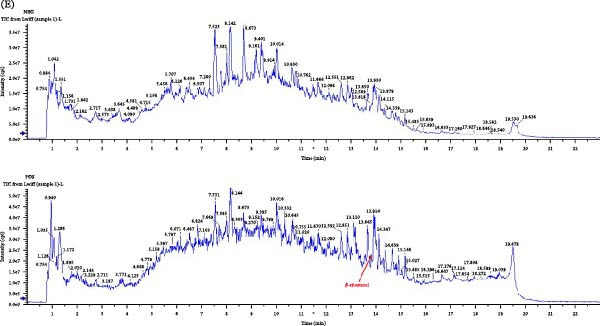

**Table 2:** The top five active ingredients in terms of TPD.

ID	Name	Average shortest path length	Betweenness centrality	Closeness centrality	Degree
MOL000098	Quercetin	2.385027	0.054858	0.419283	19
ID: 7163175	3beta‐hydroxyurs‐12‐en‐28‐syre	2.919786	0.018536	0.342491	13
ID: 64945	Ursolic acid	2.406417	0.030193	0.415556	11
MOL000358	β‐sitosterol	3.176471	0.022089	0.314815	10
MOL000006	Luteolin	3.326203	0.006865	0.300643	9

### 3.4. β‐Sitosterol in TPD Regulates PGR

**Figure 4:**
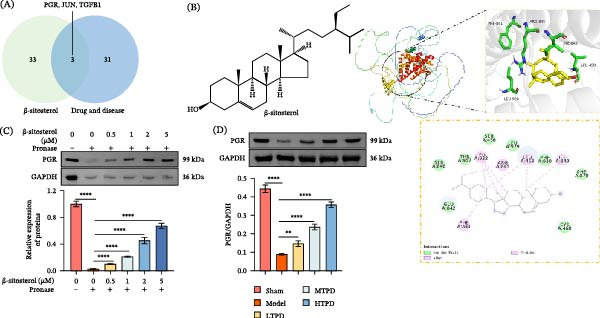
β‐sitosterol in TPD regulates PGR. (A) Venn diagram visualizing the intersection of β‐sitosterol targets and common targets of the disease and drug. (B) Chemical structure of β‐sitosterol, and molecular docking analysis of β‐sitosterol and PGR. (C) DARTS detection of β‐sitosterol binding to PGR. (D) Western blot detection of PGR expression in fallopian tube tissues. *n* = 5 rats per group.  ^∗^ *p*  < 0.05,  ^∗∗^ *p*  < 0.01,  ^∗∗∗^ *p*  < 0.001, and  ^∗∗∗∗^ *p*  < 0.0001.

Based on the results of a topological analysis, the targets of β‐sitosterol were combined to take the intersection and narrowed down to PGR, JUN, and TGFB1 (Figure 4A). Among them, PGR was associated with infertility [44]. Molecular docking simulation showed that the binding energy of β‐sitosterol to PGR was −6.8, less than −5, indicating binding (Figure 4B). DARTS results showed a decrease in PGR degradation as the concentration of β‐sitosterol increased in the presence of pronase (Figure 4C). This indicates the binding of β‐sitosterol to PGR. The effect of TPD on the PGR expression was further examined. PGR was reduced in the Model group compared to the Sham group (Figure 4D). TPD treatment increased the expression of PGR in the fallopian tube, and the effect was more obvious at high concentrations. The aforementioned data suggest that β‐sitosterol in TPD can bind to PGR and regulate its protein level.

### 3.5. PGR Inhibits Tubal Epithelial Cell Inflammation and Increases Cell Viability and Ciliary Beating Frequency

**Figure 5:**
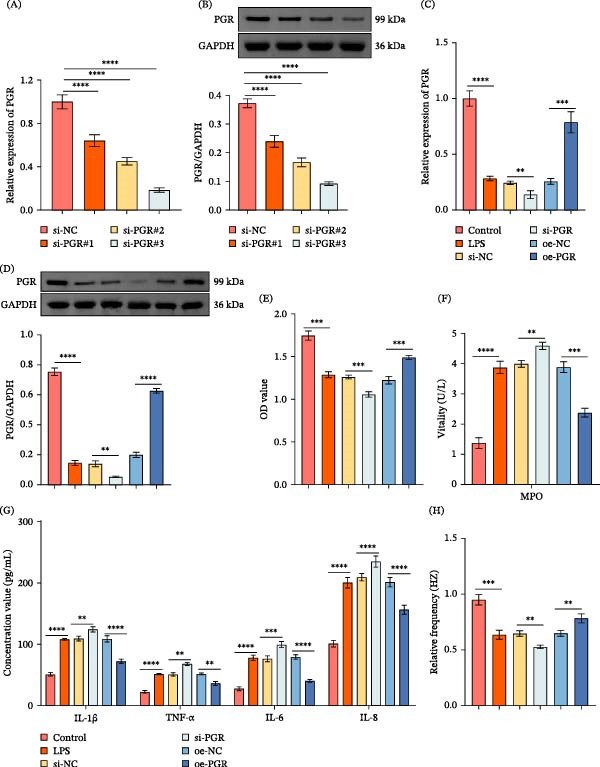
PGR inhibits tubal epithelial cell inflammation and increases cell viability and ciliary beating frequency. (A, C) RT‐qPCR assay for PGR expression. (B, D) Western blot assay for PGR expression. (E) CCK8 assay for cell viability. (F) Biochemical assay for MPO level. (G) ELISA assay for cell serum IL‐1β, IL‐6, IL‐8, and TNF‐α. (H) Ciliary beating frequency assay. *n* = 3 biological replicates.  ^∗^ *p*  < 0.05,  ^∗∗^ *p*  < 0.01,  ^∗∗∗^ *p*  < 0.001, and  ^∗∗∗∗^ *p*  < 0.0001.

To investigate the mechanism by which PGR affects tubal epithelial cell inflammation, the cells were transfected with si‐NC, si‐PGR#1, si‐PGR#2, or si‐PGR#3 to silence PGR. The results showed a high silencing efficiency of PGR after si‐PGR treatment, with si‐PGR#3 having the most pronounced effect, which was used for subsequent studies (Figure 5A,B). Compared to the control group, PGR was decreased in the LPS group (Figure 5C,D). Subsequent transfection with si‐PGR led to a further reduction, while transfection with oe‐PGR increased. Compared with the control group, cell viability was reduced, and the levels of inflammatory factors and MPO were increased in the LPS group (Figure 5E–G). Silencing of PGR further promoted an LPS‐induced decrease in cell viability as well as elevated levels of inflammatory factors and MPO. Overexpression of PGR reversed the LPS‐induced decrease in cell viability and the elevated levels of inflammatory factors and MPO. In addition, inhibiting PGR reduced the ciliary beating frequency in the LPS group (Figure 5H). In contrast, the decrease in ciliary beating frequency in the LPS group was reversed by the overexpression of PGR. The aforementioned data suggest that PGR is involved in the regulation of LPS‐induced damage and inflammation in fallopian tube epithelial cells.

### 3.6. β‐Sitosterol Acts Directly on PGR to Inhibit Tubal Epithelial Cell Inflammation and Increase Cell Viability and Ciliary Beating Frequency

**Figure 6:**
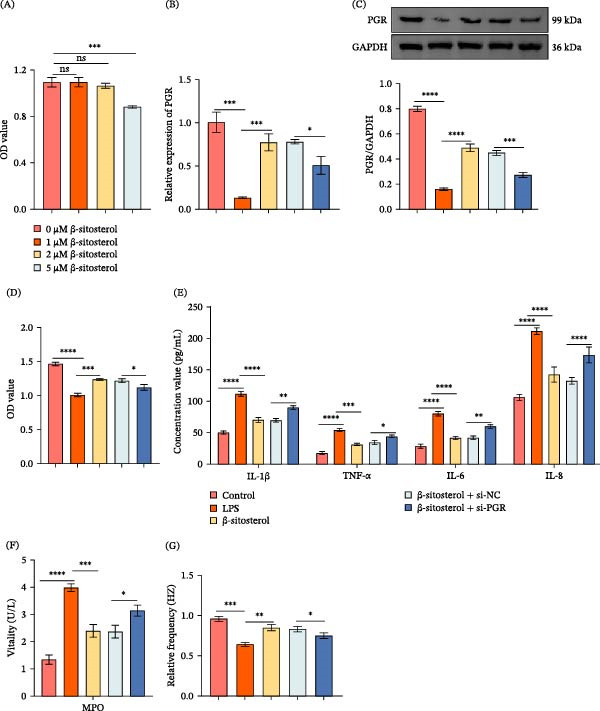
β‐sitosterol acts directly on PGR to inhibit tubal epithelial cell inflammation and increase cell viability and ciliary beating frequency. (A) Normal cells were treated with or without β‐sitosterol (1, 2, and 5 μM), and cytotoxicity was detected by CCK8. (B, C) PGR expression was detected by RT‐qPCR and western blot. (D) Cell viability was detected by CCK8. (E) ELISA was performed to detect IL‐1β, IL‐6, IL‐8, and TNF‐α in the cell supernatant. (F) Biochemical assay was performed to detect the level of MPO. (G) Detection of ciliary beating frequency in the cell supernatant. *n* = 3 biological replicates.  ^∗^ *p*  < 0.05,  ^∗∗^ *p*  < 0.01,  ^∗∗∗^ *p*  < 0.001, and  ^∗∗∗∗^ *p*  < 0.0001.

We first investigated the effect of β‐sitosterol on the toxicity of tubal epithelial cells. There was no significant difference in the cell viability of tubal epithelial cells between 1 and 2 μM of β‐sitosterol (Figure 6A). Cell viability was reduced by 5 μM of β‐sitosterol. Therefore, 2 μM was chosen for subsequent experiments. Next, to investigate whether β‐sitosterol affects tubal epithelial cell inflammation through PGR, we transfected tubal epithelial cells with si‐NC or si‐PGR plasmids and treated them with β‐sitosterol. In comparison to the control group, PGR levels were significantly lower in the LPS group and elevated after β‐sitosterol treatment (Figure 6B,C). Silencing of PGR reversed the effect of β‐sitosterol on PGR expression. Compared with the control group, cell viability was reduced in the LPS group and elevated after β‐sitosterol treatment (Figure 6D). Silencing of PGR reversed the effect of β‐sitosterol on cell viability. The expression of inflammatory factors and MPO was elevated in the LPS group compared with the Control group, while the levels of these factors were reduced after β‐sitosterol treatment (Figure 6E,F). Silencing of PGR reversed the effect of β‐sitosterol on cellular inflammation. Ciliary beating frequency was reduced in the LPS group compared with the control group and elevated after β‐sitosterol treatment (Figure 6G). Silencing PGR reversed the effect of β‐sitosterol on the ciliary beating frequency. The aforementioned results indicate that β‐sitosterol affects LPS‐induced damage and inflammation in fallopian tube epithelial cells by regulating PGR.

### 3.7. β‐Sitosterol Treats Tubal Infertile Rats via PGR

**Figure 7:**
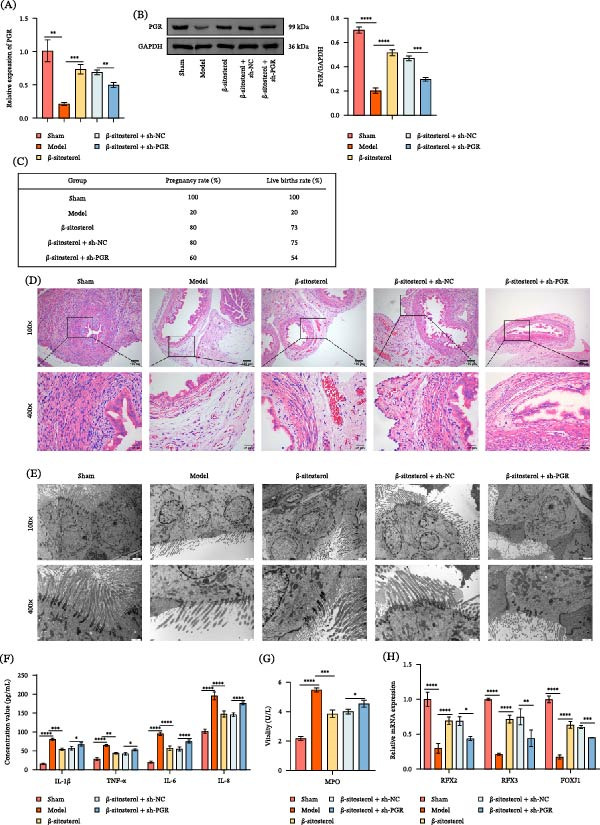
β‐sitosterol treats tubal infertile rats via PGR. (A, B) RT‐qPCR and western blot detection of PGR expression in oviductal tissues. (C) Conception rate and live birth rate statistics. (D) HE staining observation of oviductal tissues. (E) Electron microscopy observation of morphological changes of oviductal tissues. (F) ELISA detection of IL‐1β, IL‐6, IL‐8, and TNF‐α in serum. (G) Biochemical detection of MPO level. (H) RT‐qPCR to detect the expression of RFX2, RFX3, and FOXJ1. *n* = 5 rats per group.  ^∗^ *p*  < 0.05,  ^∗∗^ *p*  < 0.01,  ^∗∗∗^ *p*  < 0.001, and  ^∗∗∗∗^ *p*  < 0.0001.

After demonstrating that β‐sitosterol can up‐regulate PGR in vitro to affect tubal epithelial cell inflammation, we established a rat model of tubal infertility and intervened with β‐sitosterol and sh‐PGR. β‐sitosterol treatment of tubal infertile rats resulted in an increase in PGR expression in tissues, which was reversed by sh‐PGR (Figure 7A,B). β‐sitosterol significantly increased the conception rate and live birth rate in tubal infertile rats (Figure 7C). This was reversed by sh‐PGR. In addition, HE staining and electron microscopy showed that β‐sitosterol attenuated tubal epithelial damage in oviductally infertile rats (Figure 7D,E). sh‐PGR treatment reversed the effects of β‐sitosterol in tubal infertile rats. Levels of IL‐1β, IL‐6, IL‐8, TNF‐α, and MPO were reduced by β‐sitosterol treatment in tubal infertile rats (Figure 7F,G). RFX2, RFX3, and FOXJ1 were elevated by β‐sitosterol treatment. β‐sitosterol’s effects on these factors were reversed by sh‐PGR (Figure 7H). The aforementioned results indicate that β‐sitosterol alleviates infertility symptoms in tubal infertile rats by regulating PGR.

## 4. Discussion

Tubal infertility is one of the major causes of female infertility [45]. The process of egg and sperm transport depends mainly on the activity of cilia of tubal epithelial cells [46]. In the current study, we demonstrated that TPD and its active ingredient, β‐sitosterol, could reduce tubal epithelial cell damage and inflammation and improve conception and live birth rates in tubal infertile rats. Our results provide a research basis for the treatment of tubal infertility with TPD.

TCM treatment of tubal infertility has been based on activating blood circulation and removing blood stasis in the past [47, 48]. According to the hollow tubal structure of the fallopian tube, some medical practitioners believe that it is appropriate to dredge the fallopian tube, and better results have been achieved with the addition of drugs to promote the circulation of blood vessels [25]. We proposed a new formula, TPD, by combining the resolution of blood stasis and the clearing of collaterals. The results of in vivo experiments showed that TPD could inhibit the level of inflammatory factors, improve the damage of tubal epithelial cells, and increase the expression of RFX2 and RFX3, which are related to the formation of cilia, and FOXJ1, which is the main regulator of cilia movement in rats with tubal infertility. In vitro experimental results revealed that serum containing TPD markedly enhanced cell viability and ciliary beat frequency induced by LPS, while suppressing the LPS‐triggered increases in inflammatory factor levels and MPO activity. These results indicated that TPD had a better therapeutic effect on tubal infertility.

Network pharmacology can provide new ideas and methods for the study of TCM compounding, which can systematically analyze the components and targets of TCM compounding and contribute to a more comprehensive understanding of the mechanism of action of TCM [49, 50]. In this study, we predicted the candidate compounds and targets of TPD for the treatment of tubal infertility by the network pharmacology strategy and obtained 139 active compounds and 34 shared targets. An ingredient‐target‐gene‐disease network was constructed by combining the networks of active ingredients and disease‐causing genes. These results suggest that through the research method of network pharmacology, the effects of TPD and its key ingredients on tubal inflammatory infertility can be deeply explored, and new research avenues can be provided to improve the therapeutic efficacy.

We screened the key ingredient, β‐sitosterol, of TPD from the results of network pharmacology. β‐sitosterol is the most abundant phytosterol with anti‐inflammatory, antioxidant, and antipyretic effects [51], and it can inhibit the development of hepatocellular carcinoma [52]. Alfalfa seed oil can treat acrylamide‐induced infertility in rats, and β‐sitosterol is the active ingredient in alfalfa seed oil [53]. β‐sitosterol is efficacious in several models of inflammation. β‐sitosterol ameliorates inflammation in adipocytes of rats induced by obesity [54], attenuates mammary inflammation [55], and alleviates dextran sodium sulfate‐induced colitis [56]. In our study, we found that β‐sitosterol inhibited the levels of inflammatory factors and promoted tubal epithelial cell viability and ciliary beating frequency. In addition, in vivo experiments showed that β‐sitosterol could increase the conception rate and live birth rate of tubal infertile rats and improve oviduct injury and inflammation. For network pharmacology screening, quercetin, 3beta‐hydroxyurs‐12‐en‐28‐syre, ursolic acid, and luteolin, among the top five for TPD, have not been further explored in this study, which is a limitation of our study. We will conduct a comparison and mechanistic validation of β‐sitosterol with compounds such as quercetin in future studies to further support this strategy.

Based on the results of a topological analysis, three targets, JUN, TGFB1, and PGR, were screened by taking the intersection of β‐sitosterol targets. The three genes were searched by GeneCards [31], and the diseases associated with JUN were mainly breast cancer and cancers of the blood system. Diseases associated with TGFB1 include Camurati–Engelmann disease and inflammatory bowel disease, immunodeficiency, and encephalopathy. PGR is a key mediator of the reproductive and physiological functions of progesterone (P4). Studies have demonstrated that PGR mediates the roles of P4 in reproductive physiology, including endometrial differentiation, ovulation, embryo implantation, and successful embryonic development [57]. Specifically, P4 suppresses excessive proliferation of endometrial epithelial cells through PGR [58]. Single‐cell RNA sequencing has revealed that PGR deficiency triggers aberrant upregulation of pro‐inflammatory genes in mouse endometrial epithelial cells, indicating its anti‐inflammatory regulatory function [59]. Notably, PGR plays a central role in mammalian oviduct function, where it is specifically highly expressed at the base of motile cilia in mouse oviduct ciliated cells, participating in the regulation of oocyte transport, ovulation, and developmental competence [60]. Further studies show that PGR alleviates ovulation‐associated inflammatory responses by suppressing gonadotropin surge‐induced Ptgs2 expressions [61]. In this study, we first reported that PGR overexpression significantly inhibits LPS‐induced declines in oviduct epithelial cell viability and ciliary beating frequency, while reversing elevated inflammatory cytokine levels and abnormal MPO activity. These findings not only confirm the critical role of PGR in regulating oviduct inflammation but, more importantly, reveal the potential therapeutic value of targeting the PGR pathway to improve inflammatory infertility associated with oviduct dysfunction. Therefore, we concluded that PGR is a promising target for tubal inflammatory infertility.

We identified PGR as a direct binding partner of β‐sitosterol in TPD using molecular docking [62] and the DARTS method [63]. Our results suggest that β‐sitosterol interacts with PGR to promote its expression, which in turn inhibits tubal epithelial cell inflammation and promotes cilia formation. Silencing of PGR reversed the effects of β‐sitosterol on tubal infertile rats and tubal epithelial cells. These studies also suggest that PGR plays a key role in the treatment of tubal infertility by TPD and its active ingredient, β‐sitosterol.

Nevertheless, this study has the following limitations on data and models. The database used in this study was derived from human data, while experimental validation employed a rat model. To address this limitation, subsequent research will prioritize verification using human‐derived biological systems, including clinical specimen analyses and in vitro experiments employing human cell models, to confirm the translational and clinical relevance of our findings.

## 5. Conclusion

In summary, the present study demonstrated for the first time that TPD and its active ingredient, β‐sitosterol, play a key role in cilia movement and inflammation in tubal infertility, which is regulated by promoting the expression of PGR. These findings suggest that TPD and its active ingredient, β‐sitosterol, have a promising clinical application in the treatment of tubal infertility.

## Author Contributions

Liang Shao contributed to conceptualization, data curation, validation, writing of the original draft, and review. Nansu Wang, Yan Yan, Qiongfang Tan, and Yuying Huang contributed to formal analysis, investigation, and software. Qin Wu, Yali Tan, Yue Zhu, and Yaqiong Xia contributed to validation and methodology. Ling Liu contributed to conceptualization, funding acquisition, project administration, supervision, and review.

## Funding

This work was supported by the Scientific Research Project of Hunan Provincial Department of Education (Grant 24C1173), the Sixth Batch of National Outstanding Clinical Talents in Traditional Chinese Medicine Training Program by the State Administration of Traditional Chinese Medicine (SATCM Document on Personnel and Education No. 256 [2025]), the Hunan Provincial Department of Science and Technology’s Huxiang Youth Talent Project, the Young Shennong Scholar Program of the Hunan Provincial Health Commission, the Research Project of Hunan Provincial Health Commission (Grant W20243142).

## Disclosure

All authors have read and approved the final manuscript.

## Ethics Statement

All experimental procedures and animal handling were performed with the approval of Hunan SJA Laboratory Animal Co., Ltd. (Number IACUC‐SJA‐LL‐2023012), in accordance with the National Institutes of Health Guide for the Care and Use of Laboratory Animals, and studies involving laboratory animals follow the ARRIVE guidelines.

## Conflicts of Interest

The authors declare no conflicts of interest.

## Data Availability

The datasets used and analyzed during the current study are available from the corresponding author upon reasonable request.
